# A Novel Prognostic Four-Gene Signature of Breast Cancer Identified by Integrated Bioinformatics Analysis

**DOI:** 10.1155/2022/5925982

**Published:** 2022-02-27

**Authors:** Xiaoyu Zhao, Huimin Yan, Xueqing Yan, Zhilin Chen, Rui Zhuo

**Affiliations:** ^1^Medical College, Xuchang University, Xuchang, 461000, China; ^2^Department of Pediatrics, The First Medical Center of PLA General Hospital, Beijing, 100000, China; ^3^State Key Laboratory of Systematic and Evolutionary Botany, Institute of Botany, Chinese Academy of Sciences, Beijing, 100000, China; ^4^Department of Breast and Thoracic Oncological Surgery, The First Affiliated Hospital of Hainan Medical University, Haikou 570102, China; ^5^Department of Breast Surgery, Guilin TCM Hospital of China, Guilin 5410022, China

## Abstract

Molecular analysis facilitates the prediction of overall survival (OS) of breast cancer and decision-making of the treatment plan. The current study was designed to identify new prognostic genes for breast cancer and construct an effective prognostic signature with integrated bioinformatics analysis. Differentially expressed genes in breast cancer samples from The Cancer Genome Atlas (TCGA) dataset were filtered by univariate Cox regression analysis. The prognostic model was optimized by the Akaike information criterion and further validated using the TCGA dataset (*n* = 1014) and Gene Expression Omnibus (GEO) dataset (*n* = 307). The correlation between the risk score and clinical information was assessed by univariate and multivariate Cox regression analyses. Functional pathways in relation to high-risk and low-risk groups were analyzed using gene set enrichment analysis (GSEA). Four prognostic genes (*EXOC6*, *GPC6*, *PCK2*, and *NFATC2*) were screened and used to construct a prognostic model, which showed robust performance in classifying the high-risk and low-risk groups. The risk score was significantly related to clinical features and OS. We identified 19 functional pathways significantly associated with the risk score. This study constructed a new prognostic model with a high prediction performance for breast cancer. The four-gene prognostic signature could serve as an effective tool to predict prognosis and assist the management of breast cancer patients.

## 1. Introduction

In 2020, over 2.26 million breast cancer cases were diagnosed, accounting for 11.7% of total cancer cases in that year. Breast cancer as one of the most frequently diagnosed cancers is also a major cause of death in women. According to the Global Cancer Observatory (GCO) statistics, more than 1.4 million breast cancer cases were newly diagnosed in China in 2020, accounting for 10.3% of all cancer incidences [1]. Molecular diagnosis subdivides breast cancer into five subtypes, namely, basal-like, HER2, luminal A, luminal B, and normal-like. Specific therapeutic treatment of breast cancer varies with different subtypes and stages. Here, the use of molecular prognostic biomarkers in clinical practice could help optimize treatment and avoid unnecessary adjuvant treatment.

Compared with traditional prognostic factors such as tumor size, lymph nodes, estrogen receptor (ER), and progesterone receptor (PR), molecular prognostic biomarkers show an obvious advantage in guiding clinical decision-making for managing breast cancer patients [2]. For example, as one of the most commonly used commercial genetic prognostic tests, Oncotype DX has also been proven to be an effective tool to help predict the possibility of disease recurrence and decision-making for adjuvant chemotherapy [3–5]. A phase 3 trial SWOG-8814 demonstrates encouraging results in deciding whether tamoxifen-treated patients should accept chemotherapy by recurrence score [3].

Currently, seven types of prognostic signatures (Oncotype DX, MammaPrint, Prosigna/PAM50, EndoPredict, Breast Cancer Index, Mammostrat, and IHC4) have been included in the American Society of Clinical Oncology (ASCO) guidelines (2017 edition) and National Comprehensive Cancer Network (NCCN) guidelines [6–8]. Only Oncotype DX and MammaPrint provide treatment guidance for ER/PR-positive and HER2-negative patients [7]; however, patients with intermediate recurrence score calculated by Oncotype DX may not necessarily benefit from adjuvant chemotherapy. According to the guideline of ASCO and NCCN, Oncotype DX and MammaPrint cannot precisely determine the treatment of HER2-positive or triple-negative breast cancer [7, 8].

Genetic signatures play a significant role in predicting prognosis and deciding treatment strategies for cancer patients. Based on substantial clinical genomic data, deep genetic information can be explored through bioinformatics analysis. In this study, we identified a crucial gene cluster from the public genomic database and established a prognostic signature for breast cancer applying bioinformatics analysis.

## 2. Materials and Methods

### 2.1. Data Source

**Figure 1:**
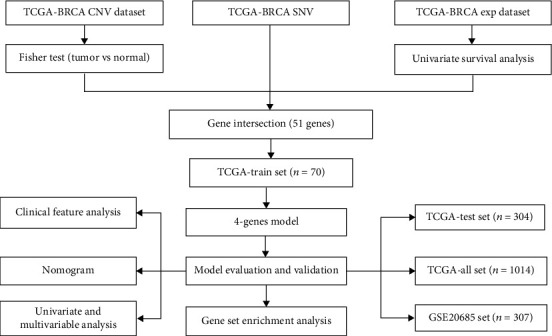
The workflow of the development and validation of the new prognostic model. 1014 samples from TCGA and 307 samples from GEO were used for analysis.

Workflow of developing the prognostic model is presented in Figure 1. The dataset of breast cancer for extracting RNA-seq, copy number variation (CNV), single nucleotide variation (SNV), and clinical follow-up information was downloaded from The Cancer Genome Atlas (TCGA) database (https://cancergenome.nih.gov/). The dataset of breast cancer with mRNA expression profiles (GSE20685) and corresponding clinical data with survival information was obtained from the Gene Expression Omnibus (GEO) database (http://www.ncbi.nlm.nih.gov/gds/?term=).

### 2.2. Data Preprocessing

Samples without clinical follow-up information and survival information were excluded. For RNA-seq dataset from TCGA, genes with a value of transcripts per million (TPM) lower than 1.0 were eliminated. According to the annotation files, probes in the GSE20685 dataset were converted to gene symbols. After excluding probes matching multiple genes, multiple symbols matching a gene were kept and calculated for the median of gene expression value. After data preprocessing, 1014 TCGA samples and 307 samples from GSE20685 were obtained. The detailed clinical information of all samples is shown in Supplementary Table S1.

### 2.3. Identification of Differentially Expressed Genes

CNV segments with an absolute value of segment_mean ≥ 0.2 were included in the following analysis. Each CNV segment of the samples (cancer and normal) was subjected to the chi-square test. False discovery rate (FDR) was calculated using the multtest R package. CNV segments with a FDR < 0.05 were mapped and converted to differential expressed genes using BEDTools [9]. The correlation between mRNA data and survival data was analyzed by the univariate Cox regression model in the R package. Differential expressed genes with *p* < 0.05 were filtered. In addition, genes showing SNV data with a mutation rate higher than 1% were identified by the MuTect tool. Genes in the intersection of CNV, SNV, and mRNA data were extracted as differentially expressed genes.

### 2.4. Dataset Processing for Establishing a Prognostic Model

A total of 1014 samples from the TCGA dataset were divided into the training group and the test group with a ratio training group : test group = 7 : 3. To ensure model stability, the samples were grouped by randomized sampling for 100 times. The division was performed based on the following conditions: (a) balanced distribution of age, sex, clinical follow-up time, and death rate between the two groups; (b) similar quantity of samples of binary classification after clustering expression profile. Divisions of the training group (710 samples) and the test group (304 samples) are displayed in Supplementary Table S2. There was no statistical difference (*p* > 0.05) between the two groups after the chi-square test.

### 2.5. Identification of Prognostic Signature within the Training Dataset

In the training dataset, differentially expressed genes significantly associated with clinical features were identified through univariate Cox regression in the R package. The multivariate Cox regression model and stepAIC in the R package were further applied to optimize the prognostic model. The simplified model with the lowest value of Akaike information criterion (AIC) was considered as the prognostic signature.

### 2.6. Calculation and Classification of Risk Score

The risk score of each sample was calculated by the prognostic model, and the prognostic signature was evaluated with receiver operating characteristic (ROC) curve. The timeROC in the R package was applied to assess ROC, and the area under ROC curve (AUC) was calculated to reflect the effectiveness of the prognostic signature. *z* − score = 0 is the cut-off for sample categorization into the low-risk group and the high-risk group.

### 2.7. Evaluating the Effectiveness of Prognostic Signature

The consistence of the signature of the test dataset was evaluated by comparing the performance of the test dataset with the training dataset. The independent dataset GSE20685 was chosen as the validation dataset for further validation. In the test dataset and validation dataset, the correlation of prognostic signature and clinical information including age, stage (I, II, III, and IV), pathological stage (T, N, and M stages), and status (PR status, ER status, and HER2 status) was analyzed by univariate and multivariable Cox regressions, ROC, and Kaplan–Meier survival curves.

### 2.8. Analyzing the Correlation between Risk Score and Functional Pathways

The correlation between the risk score and the Kyoto Encyclopedia of Genes and Genomes (KEGG) pathways was investigated. Single-sample gene set enrichment analysis (ssGSEA) in the R package was applied to analyze the gene expression profile of each sample [10, 11], and the ssGSEA score in different functional pathways of each sample was calculated to assess the correlation between KEGG pathways and the risk score. When the ssGSEA score > 0.25, KEGG pathways were defined as having a correlation with the risk score.

## 3. Results

### 3.1. Identification of Differentially Expressed Genes

**Figure 2:**
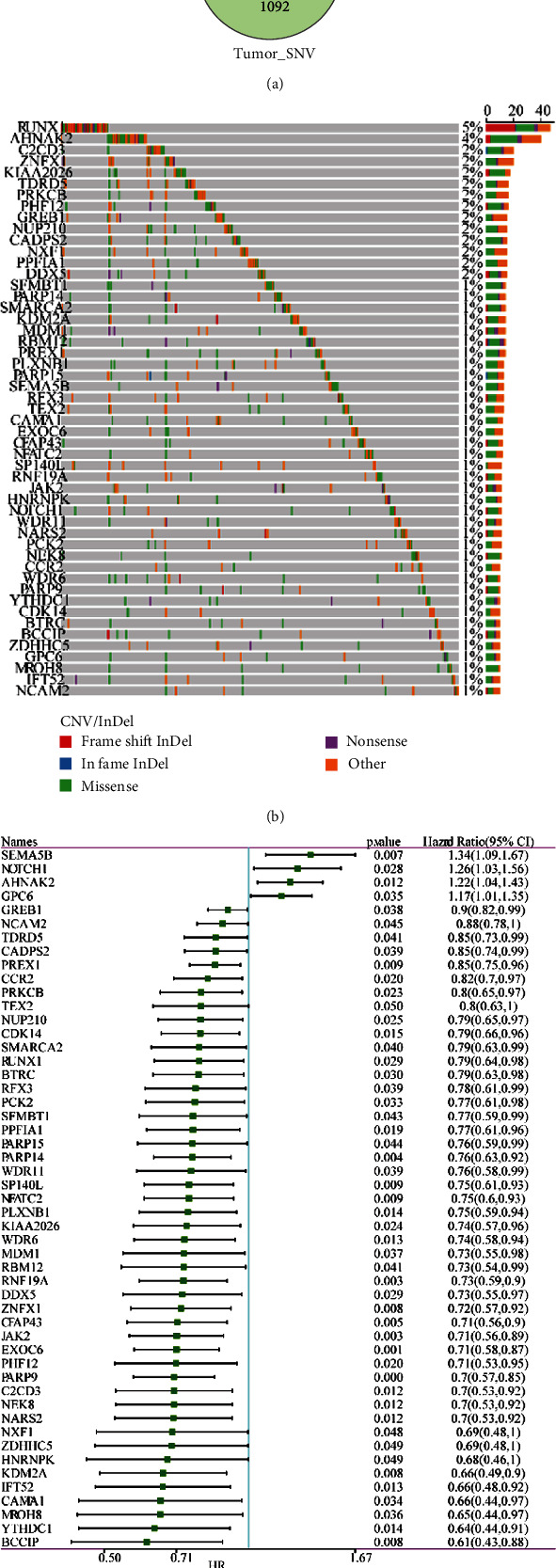
Identification of 51 differential expressed genes. (a) Venn diagram of 51 differentially expressed genes. SigCox_genes represent the genes outputted using univariate Cox regression analysis. (b) The distribution of the mutation pattern of 51 genes. Five types of mutations (frame shift indel, in frame indel, missense, nonsense, and other types) were listed. (c) Univariate Cox regression analysis of 51 differential expressed genes. HR: hazard ratio; CI: confidential interval.

After analyzing the CNV dataset of 1014 cancer samples from TCGA, 5695 differential genes were filtered by the chi-square test (FDR < 0.05, Supplementary Table S3). 3118 genes with a mutation rate greater than 1% were screened based on SNV data (Supplementary Table S4). Furthermore, 1265 differential expressed genes were screened by associating the mRNA data with survival data of 1014 samples using univariate Cox regression (*p* < 0.05, Supplementary Table S5). In Figure 2(a), 51 genes in the intersection of the screened CNV dataset, CNV dataset, and mRNA dataset were defined as differentially expressed genes (Supplementary Table S6).  Figure 2(b) shows the mutation information of these genes including mutation distribution, types, and proportion. A great majority of missense and other types of mutations can be found, and there was a great proportion (about 20%) of frame shift indel in the *RUNX1* gene. Univariate Cox regression revealed that 51 genes all had a significant correlation with survival information (Figure 2(c)). Only 4 genes (*SEMA5B*, *NOTCH1*, *AHNAK2*, and *GPC6*) showed a hazard rate (HR) > 1, indicating a significant relation between higher expression and worse prognosis (*p* < 0.05) (Figure 2(c)). The remaining 47 genes were closely related to lower expression and worse prognosis (*p* < 0.05) (Figure 2(c)).

### 3.2. Construction and Validation of the Four-Gene Prognostic Signature

A total of 1014 samples were divided into the training dataset (710 samples) and the test dataset (304 samples) by randomized sampling (Table S2), without statistical difference (*p* > 0.05). In the training dataset, 6 out of 51 differential genes were detected by univariate Cox regression (*p* < 0.05, Supplementary Table S7). The six genes were used to construct a prognostic signature and further simplified by the stepAIC method. Finally, four genes, *EXOC6*, *GPC6*, *PCK2*, and *NFATC2*, were included in the prognostic signature. Risk score was defined as follows:

(1) $$\begin{array}{c} \text{Risk score}=-0.242*\text{EXOC}6+0.255*\text{GPC}6-0.227*\text{PCK}2-0.288*\text{NFATC}2. \end{array}$$

**Figure 3:**
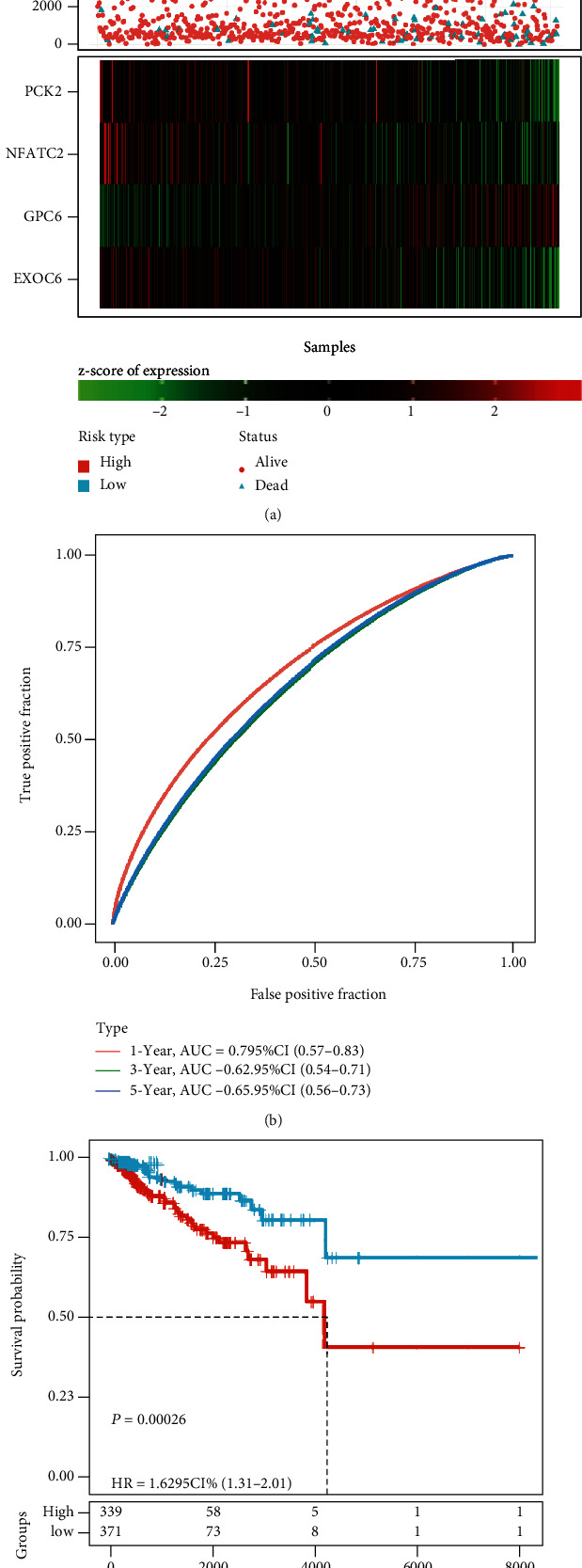
Construction of the four-gene prognostic signature in the training dataset (710 samples). (a) Risk score (converted as *z*-score) of all samples in the training dataset. Survival status (alive and dead) of 710 samples. Gene expression of prognostic genes *PCK2*, *NFATC2*, *GPC6*, and *EXOC6*. Red and green colors represented high and low expressions, respectively. (b) ROC curve of 1-year, 3-year, and 5-year survival, with AUC of 0.70, 0.62, and 0.65, respectively. (c) Kaplan–Meier survival curves of high-risk and low-risk groups classified by the four-gene signature (95%CI = 1.31 − 2.01, *p* < 0.001). HR: hazard ratio; CI: confidential interval.

According to the mRNA expression level, the risk score of each sample in the training dataset was determined and converted to the *z*-score for sample classification into the high-risk group (339 samples) and the low-risk group (331 samples). *z* − score = 0 was the cut-off (Figure 3). As shown in Figure 3(a), the samples were divided into two groups, and the mRNA expressions of four genes (*EXOC6*, *GPC6*, *PCK2*, and *NFATC2*) were consistent with the risk score. With the increase of risk score, the mRNA expression of *EXOC6*, *PCK2*, and *NFATC2* was downregulated, while that of *GPC6* was upregulated (Figure 3(a)). ROC analysis validated that the four-gene signature was an effective tool in predicting one-year, three-year, and five-year prognoses, with an AUC of 0.70, 0.62, and 0.65, respectively (Figure 3(b)). From the Kaplan–Meier survival curves, it could be found that the patient prognosis was significantly different between the high-risk group and the low-risk group (Figure 3(c), *p* < 0.001).

Similarly, the results in the test dataset (304 samples) and whole dataset (1014 samples) were consistent with those in the training dataset (*p* < 0.05 and *p* < 0.0001, respectively), pointing to a strong prognostic ability of the four-gene signature in differentiating patients with high risk and low risk (Supplementary Figure S1 and S2). Moreover, the robustness of the prognostic signature was evaluating using the independent dataset (GSE20685, with a total of 307 samples as the validation dataset). Likewise, the high-risk group (162 samples) and the low-risk group (145 samples) were effectively divided by the four-gene signature (*p* < 0.05, Supplementary Figure S3).

### 3.3. Correlation between the Four-Gene Prognostic Signature and Clinical Features

**Figure 4:**
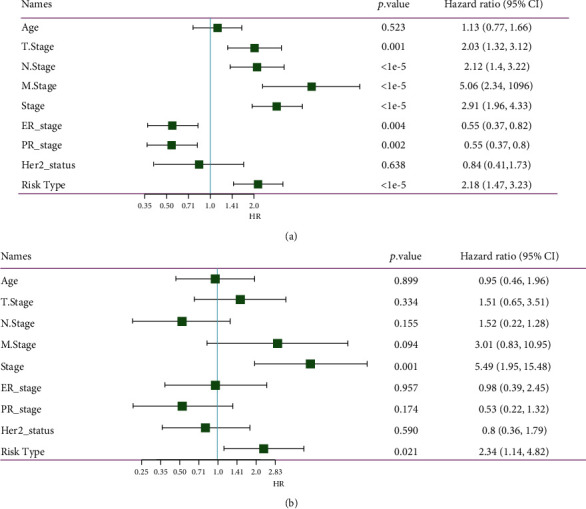
The correlation of clinical information and risk score. (a) Univariate Cox regression analysis of clinical features and risk score. (b) Multivariate Cox regression analysis of clinical features and risk score. Risk type represents high risk and low risk. HR: hazard ratio; CI: confidential interval.

**Figure 5:**
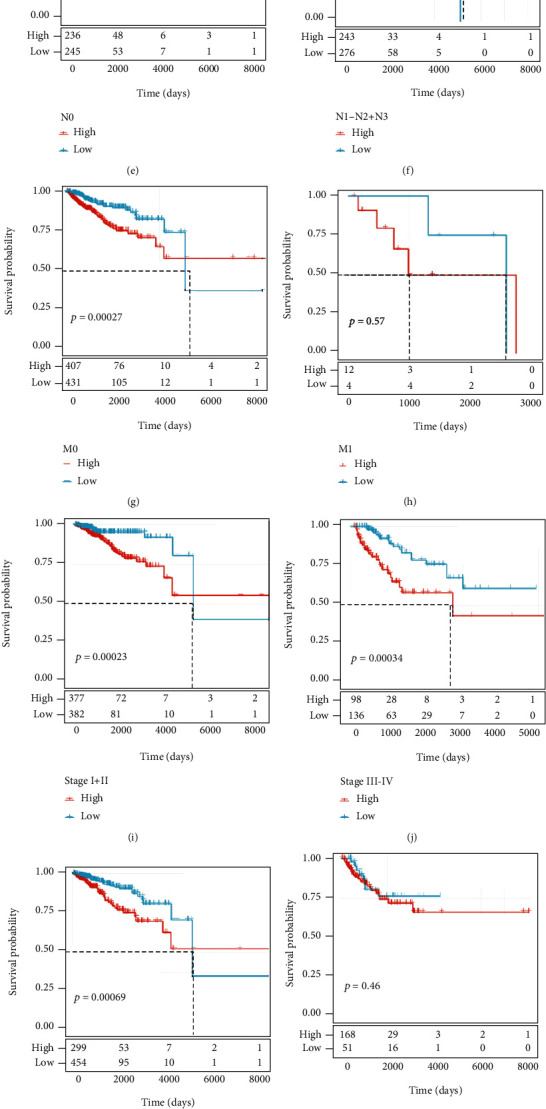
Survival analysis of clinical features including age (a, b), T stage (c, d), N stage (e, f), M stage (g, h), stage (i, j), ER status (k, l), PR status (m, n), HER2 status (o, p) in the high-risk group and the low-risk group. The red line indicates the high-risk group, and the blue line indicates the low-risk group.

**Figure 6:**
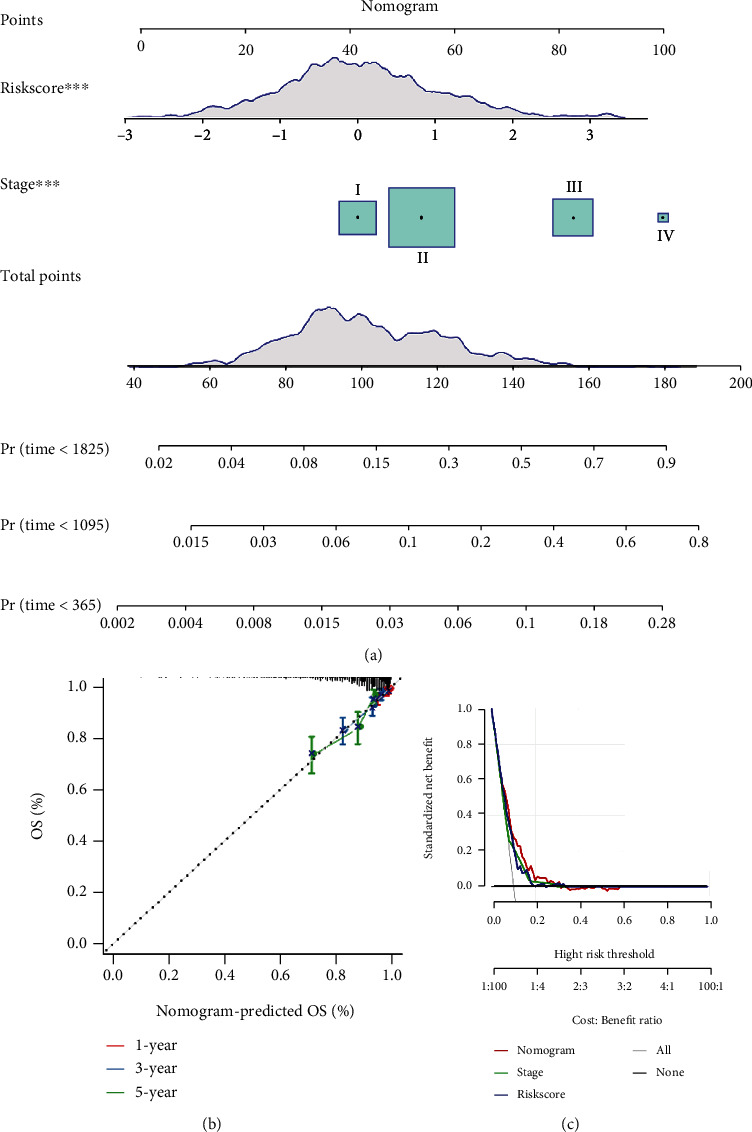
Application of four-gene prognostic signature in overall survival prediction. (a) Nomogram to predict overall survival for 1 year, 3 years, and 5 years. The horizontal axis represents the death rate. (b) The predicted overall survival and observed overall survival for 1 year, 3 years, and 5 years. (c) Decision curve analysis of nomogram, stage, and risk score. OS: overall survival.

The effectiveness of the four-gene prognostic signature was analyzed based on the correlation between the risk score and the clinical information in the TCGA dataset. Univariate Cox regression analysis showed that risk type (high risk and low risk) was associated with OS (HR = 2.18, 95%CI = 1.47–3.23, *p* < 0.00001, Figure 4(a)). Multivariate Cox regression analysis also demonstrated a significant correlation between risk type and survival (HR = 2.34, 95%CI = 1.14–4.82, *p* < 0.05, Figure 4(b)). The distribution of risk score in different clinical features manifested a significant difference of risk score in the M stage (M0 and M1), stages I to IV, ER status, PR status, HER2 status, and subtypes (*p* < 0.05, Supplementary Figure S4^1^). The survival plots showed that patients in the low-risk group in all clinical statuses all had a longer survival (Figure 5). In particular, clinical features including age, T stage, N stage, M0 stage, stages I to IV, ER-positive status, PR-positive status, and HER2-negative status could be clearly divided into the high-risk group and the low-risk group by the prognostic signature (*p* < 0.05, Figure 5), but the risk score system was not sensitive to M1 status, ER-negative, PR-negative, or HER2-positive samples. Moreover, we compared the clinical difference between the high-risk group and the low-risk group. Although there was no significant difference of T, N, and M stages between the two groups, a significant difference of stages I to IV was detected (*p* < 0.05, Supplementary Figure S5). Additionally, a nomogram was developed based on the risk score and cancer stage (Figure 6(a)). The predicted death rate was positively related to the survival time and total points (Figure 6(a)). The predicted survival of 1 year, 3 years, and 5 years was adjusted according to the observed survival data (Figure 6(b)). Decision curve analysis (DCA) revealed that risk score was effective in OS prediction, but the nomogram showed greater advantages (Figure 6(c)).

### 3.4. Correlation between Risk Score and Functional Pathways

**Figure 7:**
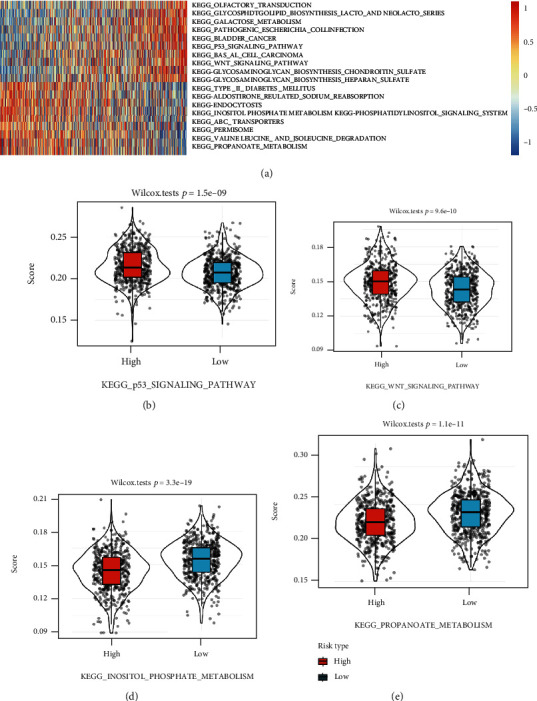
The correlation between risk score and functional pathways. (a) Heat map of 19 functional pathways related to risk score (ssGSEA score > 0.25). (b) The comparison of high-risk and low-risk groups in four pathways, p53 signaling pathway, Wnt signaling pathway, propanoate metabolism pathway, and inositol phosphate metabolism pathway.

GSEA analysis analyzed the relation between the mRNA expression and functional pathways using the TCGA dataset. The ssGSEA score of each sample was calculated to evaluate the correlation coefficient with risk score. Functional pathways with a correlation coefficient > 0.25 are shown in Figure 7(a), in which 10 pathways had a positive relation with risk score and 9 pathways had a negative relation with risk score. In particular, the p53 signaling pathway and the Wnt signaling pathway were positively related to the risk score, while the propanoate metabolism pathway and the inositol phosphate metabolism pathway were negatively related to the risk score (*p* < 0.00001, Figure 7(b)). In addition, mutation frequency and pattern were compared between the high-risk and low-risk groups (Supplementary Figure S6). Three genes (*TP53*, *PIK3CA*, and *CDH1*) showed significant difference between the two groups. The mutation frequency of *TP53* in the high-risk group was higher than that in the low-risk group, and those of *PIK3CA* and *CDH1* were lower in the high-risk group (Supplementary Figure S6).

## 4. Discussion

Prognostic signatures such as Oncotype DX and MammaPrint have been approved by the Food and Drug Administration (FDA) and commercially applied in clinical practice, but breast cancer patients had limited benefit from them [7, 8]. Currently, there is no available effective prognostic signature to guide decision-making of the treatment plan for patients with HER2-positive, node-positive, and triple-negative breast cancer (TNBC).

In the present study, we used the available data of breast cancer from two databases (TCGA and GEO) and applied a new methodology combined with CNV, SNV, and mRNA data to mine differentially expressed genes. Based on differentially expressed genes and patients' clinical information, a prognostic model was developed and further optimized with the Akaike information criterion. Finally, a four-gene prognostic signature based on *EXOC6*, *GPC6*, *PCK2*, and *NFATC2* was established, which had a high performance in classifying samples to two groups (high risk and low risk) in the test dataset and the validation dataset. Clinical features including T stage, N stage, M stage, stage, ER status, and PR status were significantly associated with risk score. The prognostic signature with only four genes involved was more clinically friendly than current commercial signatures of breast cancer.

Recent studies have proposed several prognostic signatures of breast cancer. For instance, Alsaleem et al. developed a two-gene signature (*ACSM4* and *SPDYC*) indicative of poor prognosis of TNBC [12]; Joe et al. explored a prognostic gene set with a total of 43 genes from the transcriptomic dataset of breast cancer; and Deng et al. discovered six hub genes (*CDK1*, *CCNA2*, *TOP2A*, *CCNB1*, *KIF11*, and *MELK*) associated with worse overall survival of breast cancer patients [13]. In a previous study, differentially expressed genes were screened from 235 GEO samples, and 1105 samples from TCGA served as a validation dataset [13]. In another study, using weighted gene coexpression network analysis (WGCNA), five hub genes (*CCNB2*, *FBXO5*, *KIF4A*, *MCM10*, and *TPX2*) consisted of a prognostic signature and were correlated with poor prognosis [14], but only the transcriptomic dataset was included in the study [14]. Previously, differentially expressed genes were filtered based on mRNA expression data; however, current results generated by combined analysis of CNV, SNV, and mRNA data will be more reliable and comprehensive. Furthermore, the R software package CIBERSORT, Timer, and MCPcounter were used to evaluate the infiltration score of immune-infiltrating cells in each patient and observed that CD8 T cells were significantly higher in the high-risk group than in the low-risk patients (Supplementary Figure S7). PAM50 subtype analysis showed that the 4-gene model was more suitable for predicting the prognosis in Her2 and LumB subtypes (Supplementary Figure S8).

In the four-gene prognostic signature, *EXOC6*, *GPC6*, and *NFATC2* were correlated with aggression of breast cancer. Pan cancer analysis indicated that in the expressions of four genes, at least one significant difference in the expression of these genes was observed in multiple tumors, but four genes had significant differences in the expression of breast cancer (Supplementary Figure S9). The EXOC6 protein as one of the components of the exocyst complex plays crucial role in exocytosis and is involved in intracellular content delivery. The exocyst complex is implicated in some diseases including in kidney diseases, neuropathogenesis, diabetes, and cancers [15]. *EXOC6* has been reported to be a predictive biomarker in the sensitivity evaluation of treatment with SAHA (suberoylanilide hydroxamic acid) and paclitaxel [16]. Research showed that *EXOC6* is upregulated in the paclitaxel-resistant combination synergistic cell lines [16]. Winham et al. demonstrated that the *EXOC6* expression in breast cancer cases is higher than that in control cases, thereby concluding that *EXOC6* is a predictive gene in breast cancer development [17].

*GPC6*, a member of the glypican family (six members of *GPC1*-*GPC6*), plays an important role in development and morphogenesis [18]. *GPC1* has been discovered to have a higher expression in breast cancer tissues and cells than normal breast tissues and may contribute to the progression of breast cancer [19]. *GPC6* is related to various tumors including prostate cancer [20], non-small cell lung cancer [21], colorectal cancer [22], gastric cancer [23], early stage ovarian cancer [24], nasopharyngeal carcinoma [25], and breast cancer [26]. Notably, *GPC6* promotes invasive migration through inhibiting *β*-catenin and Wnt signaling pathways and upregulating noncanonical Wnt5A signaling [26]. In the current study, the Wnt signaling pathway showed a positive correlation with risk score (Figure 7). Based on the analysis of 3951 breast cancer patients from a public database, Grillo et al. suggested that glypicans could serve as prognostic biomarkers for breast cancer patients [27] as they found that low GPC6 was correlated with longer survival time [27], which is consistent with our findings that the low-risk group had lower GPC6 expression.

*NFATC2* (also known as *NFATP* or *NFAT1*) belongs to the nuclear factor of activated T cell (NFAT) family and regulates the expression of cytokine interleukin-2 (IL-2) in activated T cells [28]. Many researches demonstrated the critical functions of *NFATC2* in cancers such as colon cancer [29], pancreatic cancer [30, 31], lung adenocarcinoma [32], melanoma [33], and other cancers [34]. Interestingly, Yiu et al. proved that NFAT binds to three regulatory elements in the *GPC6* proximal promoter and stimulates breast carcinoma invasion by inducing *GPC6* [26]. Ding et al. indicated that NFATC2 may act as a pivotal factor for OSW-1-mediated effects on cell death, tumor growth, invasion, and migration of triple-negative breast cancer [35]. Moreover, it has been unveiled that NFATC2 is negatively correlated with Stat5 and that these two transcription factors may significantly influence the progression of breast cancer [36]. However, *PCK2* gene has not been reported in breast cancer. *PCK2* encodes a mitochondrial isoform of phosphoenolpyruvate carboxykinase (PEPCK) [37]. It was demonstrated that *PCK2* was involved in the tumor proliferation of lung cancer [38–40], prostate cancer [41], and hepatocellular carcinoma [42]. We also did a protein interaction network analysis; the result showed that the four genes formed a small world, with weak links between them (Supplementary Figure S10) suggesting that the smaller bioinformatics overlap between these genes, with greater biological information between them complementary to each other.

In this study, we applied a new methodology to develop a prognostic model for breast cancer. The four-gene prognostic signature showed a satisfactory performance to some extent, except that it was not sensitive to ER-negative, PR-negative, and HER2-positive samples. In addition, we did not consider epigenetic effects as DNA methylation has been found to be correlated with particular breast cancer subtypes [43, 44]. This novel prognostic signature could be expected to guide the treatment decision-making and predict the prognosis of breast cancer patients or even promote the discoveries of new molecular drug targets. However, before that, further clinical samples and evidence should be gathered to validate these new prognostic genes.

## 5. Conclusion

In conclusion, our study developed a novel prognostic signature closely correlated with the overall survival in breast cancer. All the samples were classified into a high-risk or a low-risk group by the risk score system. In particular, the risk score was sensitive to clinical features including the tumor stage, ER-positive status, PR-positive status, and HER2-negative status. Therefore, our four-gene signature could serve as new prognostic biomarkers for breast cancer, providing a new direction for exploring new drugs or therapies of breast cancer.

## Data Availability

The dataset generated and/or analyzed during the current study is available in the [GSE20685] repository [https://www.ncbi.nlm.nih.gov/geo/query/acc.cgi?acc=GSE20685].

## Supplementary Materials

Supplementary Materials Supplementary Figure S1: validation of four-gene prognostic signature in the training dataset (304 samples). (A) Risk score (converted as *z*-score) of all samples in the training dataset. Survival status (alive and dead) of 304 samples. Gene expression of prognostic genes *PCK2*, *NFATC2*, *GPC6*, and *EXOC6*. Red and green colors represented high and low expressions, respectively. (B) ROC curve of 1-year, 3-year, and 5-year survival, with AUC of 0.72, 0.61, and 0.66, respectively. (C) Kaplan–Meier survival curves of high-risk and low-risk groups classified by the four-gene signature (95%CI = 1.04 − 1.95, *p* < 0.05). HR: hazard ratio; CI: confidential interval. Supplementary Figure S2: validation of four-gene prognostic signature in the TCGA dataset (1014 samples). (A) Risk score (converted as *z*-score) of all samples in the training dataset. Survival status (alive and dead) of 1014 samples. Gene expression of prognostic genes *PCK2*, *NFATC2*, *GPC6*, and *EXOC6*. Red and green colors represented high and low expressions, respectively. (B) ROC curve of 1-year, 3-year, and 5-year survival, with AUC of 0.70, 0.62, and 0.65, respectively. (C) Kaplan–Meier survival curves of high-risk and low-risk groups classified by four-gene signature (95%CI = 1.30 − 1.86, *p* < 0.0001). HR: hazard ratio; CI: confidential interval. Supplementary Figure S3: validation of four-gene prognostic signature in the GSE20685 dataset (307 samples). (A) Risk score (converted as *z*-score) of all samples in the training dataset. Survival status (alive and dead) of 1014 samples. Gene expression of prognostic genes *PCK2*, *NFATC2*, *GPC6*, and *EXOC6*. Red and green colors represented high and low expressions, respectively. (B) ROC curve of 1-year, 3-year, and 5-year survival, with AUC of 0.76, 0.72, and 0.66, respectively. (C) Kaplan–Meier survival curves of high-risk and low-risk groups classified by four-gene signature (95%CI = 1.05 − 2.36, *p* < 0.05). HR: hazard ratio; CI: confidential interval. Supplementary Figure S4: the distribution of different clinical features (relapse, T stage, N stage, M stage, stage, and age) in high-risk and low-risk groups in the TCGA dataset (1014 samples). ^∗^*p* < 0.05. Supplementary Figure S5: comparison of different clinical features in high-risk and low-risk groups in the TCGA dataset (1014 samples). Kruskal-Wallis test was used to compare the difference of T stage (A), N stage (B), stage (D), and subtype (I). Wilcoxon test was used to compare the difference of M stage (C), age (E), ER status (F), PR status (G), and HER2 status (H). Supplementary Figure S6. The mutation pattern of 15 genes in the high-risk group (A) and the low-risk group (B). Different colors represented different types of mutations. The right bar and percentage represented the quantity and proportion of mutations. Supplementary Figure S7: immune infiltration of risk types. A: 22 immune cell infiltration scores in tumor samples assessed by CIBERSORT. B: 6 immune cell infiltration scores in tumor samples evaluated by Timer software. C: 10 immune cell infiltration scores in tumor samples assessed by MCPcounter software. Supplementary Figure S8: prognostic ROC curve of the model in the PAM50 subtype. Supplementary Figure S9: differential expression analysis of four genes in Pan carcinoma. Supplementary Figure S10: the interaction network of 30 genes in the nearest neighbors of 4 genes. Supplementary Table S1: TCGA dataset (1014 samples) and GSE20685 dataset (307 samples) of breast cancer. RFS: recurrence-free survival; ER: estrogen receptor; PR: progesterone receptor. Supplementary Table S2: training dataset and test dataset of total 1014 samples from the TCGA dataset. RFS: recurrence-free survival; ER: estrogen receptor; PR: progesterone receptor. Supplementary Table S3: 5695 differential genes identified from the CNV dataset using the chi-square test. Supplementary Table S4: 1265 differentially expressed genes identified from the mRNA dataset using univariate Cox regression analysis. Supplementary Table S5: 3118 differential genes identified from the SNV dataset with mutation rate > 1%. Supplementary Table S6: 51 differentially expressed genes from the intersection of the CNV dataset, SNV dataset, and mRNA dataset. Supplementary Table S7: univariate Cox regression analysis of 6 differentially expressed genes in the training dataset. HR: hazard ratio; CI: confidential interval.
