# Supplementary material for: A Novel Prognostic Four-Gene Signature of Breast Cancer Identified by Integrated Bioinformatics Analysis
Source: Dis Markers. 2022 Feb 27;2022:5925982. doi: 10.1155/2022/5925982 (PMC8898848; doi:10.1155/2022/5925982)
Supplement: Supplementary Materials — Supplementary Figure S1: validation of four-gene prognostic signature in the training dataset (304 samples). (A) Risk score (converted as z-score) of all samples in the training dataset. Survival status (alive and dead) of 304 samples. Gene expression of prognostic genes PCK2, NFATC2, GPC6, and EXOC6. Red and green colors represented high and low expressions, respectively. (B) ROC curve of 1-year, 3-year, and 5-year survival, with AUC of 0.72, 0.61, and 0.66, respectively. (C) Kaplan–Meier survival curves of high-risk and low-risk groups classified by the four-gene signature (95%CI = 1.04 − 1.95, p < 0.05). HR: hazard ratio; CI: confidential interval. Supplementary Figure S2: validation of four-gene prognostic signature in the TCGA dataset (1014 samples). (A) Risk score (converted as z-score) of all samples in the training dataset. Survival status (alive and dead) of 1014 samples. Gene expression of prognostic genes PCK2, NFATC2, GPC6, and EXOC6. Red and green colors represented high and low expressions, respectively. (B) ROC curve of 1-year, 3-year, and 5-year survival, with AUC of 0.70, 0.62, and 0.65, respectively. (C) Kaplan–Meier survival curves of high-risk and low-risk groups classified by four-gene signature (95%CI = 1.30 − 1.86, p < 0.0001). HR: hazard ratio; CI: confidential interval. Supplementary Figure S3: validation of four-gene prognostic signature in the GSE20685 dataset (307 samples). (A) Risk score (converted as z-score) of all samples in the training dataset. Survival status (alive and dead) of 1014 samples. Gene expression of prognostic genes PCK2, NFATC2, GPC6, and EXOC6. Red and green colors represented high and low expressions, respectively. (B) ROC curve of 1-year, 3-year, and 5-year survival, with AUC of 0.76, 0.72, and 0.66, respectively. (C) Kaplan–Meier survival curves of high-risk and low-risk groups classified by four-gene signature (95%CI = 1.05 − 2.36, p < 0.05). HR: hazard ratio; CI: confidential interval. Supplementary Figure S4: th [file 5925982.f1.docx]

## Description of supplementary materials

Supplementary Figure S1. Validation of four-gene prognostic signature in the training dataset (304 samples). (A) Risk score (converted as z-score) of all samples in the training dataset. Survival status (alive and dead) of 304 samples. Gene expression of prognostic genes *PCK2*, *NFATC2*, *GPC6* and *EXOC6*. Red and green color represented high and low expression respectively. (B) ROC curve of 1-year, 3-year and 5-year survival, with AUC of 0.72, 0.61, and 0.66, respectively. (C) Kaplan–Meier survival curves of high-risk and low-risk group classified by the four-gene signature (95% CI = 1.04-1.95, *p* < 0.05). HR, hazard ratio. CI, confidential interval.

Supplementary Figure S2. Validation of four-gene prognostic signature in the TCGA dataset (1014 samples). (A) Risk score (converted as z-score) of all samples in the training dataset. Survival status (alive and dead) of 1014 samples. Gene expression of prognostic genes *PCK2*, *NFATC2*, *GPC6* and *EXOC6*. Red and green color represented high and low expression respectively. (B) ROC curve of 1-year, 3-year and 5-year survival, with AUC of 0.70, 0.62, and 0.65, respectively. (C) Kaplan–Meier survival curves of high-risk and low-risk group classified by four-gene signature (95% CI = 1.30-1.86, *p* < 0.0001). HR, hazard ratio. CI, confidential interval.

Supplementary Figure S3. Validation of four-gene prognostic signature in the GSE20685 dataset (307 samples). (A) Risk score (converted as z-score) of all samples in the training dataset. Survival status (alive and dead) of 1014 samples. Gene expression of prognostic genes *PCK2*, *NFATC2*, *GPC6* and *EXOC6*. Red and green color represented high and low expression respectively. (B) ROC curve of 1-year, 3-year and 5-year survival, with AUC of 0.76, 0.72, and 0.66, respectively. (C) Kaplan–Meier survival curves of high-risk and low-risk group classified by four-gene signature (95% CI = 1.05-2.36, *p* < 0.05). HR, hazard ratio. CI, confidential interval.

Supplementary Figure S4. The distribution of different clinical features (relapse, T stage, N stage, M stage, Stage, and age) in high-risk and low-risk group in TCGA dataset (1014 samples). **p* < 0.05.

Supplementary Figure S5. Comparison of different clinical features in high-risk and low-risk group in TCGA dataset (1014 samples). Kruskal-Wallis test was used to compare the difference of T stage (A), N stage (B), Stage (D), subtype (I). Wilcoxon test was used to compare the difference of M stage (C), age (E), ER status (F), PR status (G), HER2 status (H).

Supplementary Figure S6. The mutation pattern of 15 genes in high-risk group (A) and low-risk group (B). Different colors represented different types of mutations. The right bar and percentage represented the quantity and proportion of mutations.

Supplementary figure S7. Immune infiltration of RiskTypes. A: 22 immune cell infiltration scores in tumor samples assessed by CIBERSORT. B: 6 immune cell infiltration score in tumor samples evaluated by Timer software. C: 10 immune cell infiltration scores in tumor samples assessed by MCPcount software.

Supplementary figure S8. Prognostic ROC curve of the model in PAM50 subtype

Supplementary figure S9. Differential expression analysis of four genes in Pan carcinoma.

Supplementary figure S10. The interaction network of 30 genes in the nearest neighbors of 4 genes.

Supplementary Table S1. TCGA dataset (1014 samples) and GSE20685 dataset (307 samples) of breast cancer. RFS, recurrence-free survival. ER, estrogen receptor. PR, progesterone receptor.

Supplementary Table S2. Training dataset and test dataset of total 1014 samples from TCGA dataset. RFS, recurrence-free survival. ER, estrogen receptor. PR, progesterone receptor.

Supplementary Table S3. 5695 differential genes identified from CNV dataset using *Chi*-square test.

Supplementary Table S4. 1265 differentially expressed genes identified from mRNA dataset using univariate Cox regression analysis.

Supplementary Table S5. 3118 differential genes identified from SNV dataset with mutation rate > 1%.

Supplementary Table S6. 51 differentially expressed genes from the intersection of CNV dataset, SNV dataset and mRNA dataset.

Supplementary Table S7. Univariate Cox regression analysis of 6 differentially expressed genes in the training dataset. HR, hazard ratio. CI, confidential interval.
